# Characterizing Biomass Yield and Nutritional Value of Selected Indigenous Range Species from Arid Tunisia

**DOI:** 10.3390/plants10102031

**Published:** 2021-09-27

**Authors:** Mounir Louhaichi, Mouldi Gamoun, Sawsan Hassan, Mohamed A. B. Abdallah

**Affiliations:** 1 International Center for Agricultural Research in the Dry Areas (ICARDA), Tunis 1004, Tunisia; M.Gamoun@cgiar.org; 2 Department of Animal and Rangeland Science, Oregon State University, Corvallis, OR 97331, USA; abdallam@oregonstate.edu; 3 International Center for Agricultural Research in the Dry Areas (ICARDA), Amman 11185, Jordan; S.Hassan@cgiar.org

**Keywords:** biodiversity conservation, chemical composition, crude protein, livestock productivity, mineral composition, nutritive value

## Abstract

Rangelands of Tunisia show a great indigenous species diversity with considerable potential as forage for livestock. However, information on their fodder yield and quality is scanty and restricted to few species. The objective of the study was to evaluate the nutritive values of selected key perennial species based on their biomass yield, chemical composition, in vitro organic matter digestibility (IVOMD), and mineral composition. The species evaluated included four grass species (*Stipa lagascae* Roem. and Schult., *Stipa tenacissima* L., *Stipagrostis plumosa* (L.) Munro ex T. Anderson, and *Stipagrostis pungens* (Desf.) de Winter.) and eight shrub species (*Anthyllis henoniana* Coss. ex Batt., *Argyrolobium uniflorum* (Deene.) Jaub. and Spach., *Echiochilon fruticosum* Desf., *Gymnocarpos decander* Forssk., *Helianthemum kahiricum* Delile., *Helianthemum lippii* (L.) Dum. Cours., *Plantago albicans* L. and *Rhanterium suaveolens* Desf.). Results showed that shrub species contained higher concentrations of the crude protein (CP), acid detergent lignin (ADL), but lower neutral detergent fiber (aNDFom) and acid detergent fiber (ADFom) concentrations than grasses. The greatest concentration of CP was 135 g/kg DM for *R. suaveolens*. The greatest aNDFom concentration was found within the grasses with maximum of 744.5 g/kg DM in *S. plumosa*. The shrub species *E. fruticosum*, *A. uniflorum*, *P. albicans*, *G. decander*, *R. suaveolens*, and *A. henoniana* had the highest IVOMD with over 500 g/kg DM and have the potential to supply energy to livestock. Overall, the moderate to high protein, low fiber, and high in vitro digestibility measured for shrubs, suggest they have high nutritional values and can be used to enhance local livestock production.

## 1. Introduction

Rangelands cover about 27% of the world’s land surface area and comprise 70% of the world’s agricultural land area [1]. In Tunisia, rangelands comprise nearly one-quarter of the entire land cover, totaling about 5.5 million hectares, 87% of which consist of arid to semi-arid conditions [2]. The rangelands of Tunisia show a great diversity of species composition [3] and ultimately play a key role in domestic livestock feeding as a fundamental component of animal diets during grazing periods [4]. Range animal productivity depends on palatability, availability, and forage nutritive value [5]. Thus, sustainable use of these rangelands is an important element of successful livestock production.

Unfortunately, rangelands in Tunisia are facing a myriad of problems, in particular, overgrazing and droughts. Heavy grazing of natural rangelands results in a decline of species richness of highly palatable plant species [6] and destruction of native forage plants, which are then replaced either by annuals that have little forage value or by unpalatable and toxic species [7,8]. For instance, *Astragalus armatus* Willd., a long-spined species, and *Thymelaea hirsuta* (L.) Endl., a toxic and highly fibrous species are widespread in southern Tunisian rangelands that have been subject to heavy grazing [9]. The protection of rangelands to exclude livestock grazing is widely considered to be a simple and effective practice for restoring the vegetation structure in degraded arid rangelands and has been found to increase the number of palatable plant species, such as *Echiochilon fruticosum* Desf., *Helianthemum lippii* (L.) Dum. Cours., *Stipa lagascae* Roem. and Schult. and *Stipagrostis plumosa* (L.) Munro ex T. Anderson [2,10].

In Tunisia’s southeastern rangelands, protection has increased the proportion of palatable species to more than 50%, much higher than in overgrazed areas [11]. Livestock feed resources in the southern region of Tunisia include natural rangelands, crop residue and agro-industrial by-products, of which the first contribute the largest share. Despite the significant degradation of these rangelands as a result of overgrazing and climate change, they remain the predominant and most cost-effective natural source of nutrients for ruminants [12,13].

The arid areas of Tunisia, including desert rangelands, are mainly constrained by dry season feed shortages, with the lack of protein, minerals and energy being the most limiting. The fodder resources of natural rangelands are very low in total nitrogen (N) and crude protein (CP), ranging between 5–25%, as well as low digestibility potential and low mineral concentration [14]. While natural rangelands can provide sufficient feed during the rainy season, seasons of drought are normally accompanied by persistent feed shortages and a rapid decrease in palatable species with high nutritive value, thereby constraining sustainable animal production.

During the dry season, the CP concentration of mature grasses, for example, declines to 1–2% in tropical grasslands compared to wet seasons [15]. In Tunisia’s arid zones, the production of Chamaephyte shrubs, such as *Anthyllis henoniana* Coss. ex Batt., *Argyrolobium uniflorum* (Deene.) Jaub. and Spach., *E. fruticosum*, *Gymnocarpos decander* Forssk., *Helianthemum kahiricum* Delile., *H. lippii*, and *Rhanterium suaveolens* Desf. represent a significant amount of annual production. The variability of annual production is 60–80% or 50–90%, expressed as a percentage of total annual dry matter (DM) production [16,17].

Rangeland degradation based on qualitative and quantitative estimates has, in some situations, led to desertification and facilitated the introduction of non-indigenous species for rangeland restoration in Tunisia [18]. Awareness of the phylogenetic heritage of pastoral plant species has thus received increasing attention over the years, leading to the collection of major plant species from their native habitats and storing the seeds in gene banks to establish a reference collection for Tunisia’s arid and desert area indigenous species [19]. However, there is limited information about the nutritional value of these indigenous species. Since the nutritional status of available forage has a direct effect on livestock production [20], range managers must understand the nutritional dynamics of forages for the purpose of adopting strategies to maintain adequate animal growth and reproduction [21].

An evaluation of the nutritional content of forage species is generally made by measuring the content of nutrients [22]. Frequently used indicators of forage nutritive value are crude protein (CP), metabolizable energy (ME), neutral detergent fiber (aNDFom), and acid detergent fiber (ADFom) [23,24,25]. Although forage species in natural rangelands generally provide nutrients at a lower cost than concentrate feeds, they are inherently variable in their nutritive value [26]. Factors such as the forage species, degree of plant maturity, soil type and local climate all influence a plant’s nutritional value [20]. In addition, the chemical composition of forage is an important palatability factor influencing yield quantity and quality [27]. The objective of this study was to characterize and assess the extent of variation in fodder biomass and quality among key rangeland species grown in dryland environments in southern Tunisia. These findings will contribute to enhanced livestock production by supporting decisions that optimize both forage yield and nutritive value.

## 2. Results

### 2.1. Plant Growth and Yield Attributes

**Figure 1:**
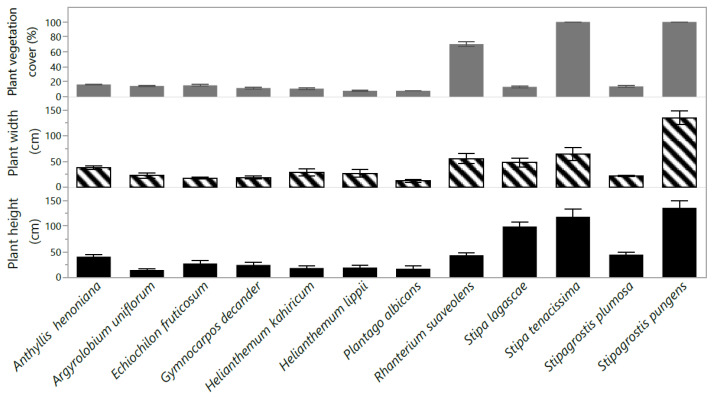
Plant height (cm), width (cm), and vegetation cover (%) of twelve native perennial species from arid rangelands of southern Tunisia. Values are the means ± standard error (SE) from 3 replicates (means ± SE).

This Plant height, width, and vegetation cover varied (*p* < 0.05) by species (Figure 1). Plant height, width, and vegetation cover were highest in the grass species *Stipagrostis Pungens* (Desf.) de Winter. (height: 136 cm, width: 221 cm, vegetation cover 23.9%). *A. uniflorum* had the lowest height of 14 cm. The shrub species *Plantago Albicans* L. had the least plant width (14 cm). The vegetation cover of shrub species *P. albicans* and *H. lippii* was the lowest (~14%) among all the studied species (Figure 1).

**Figure 2:**
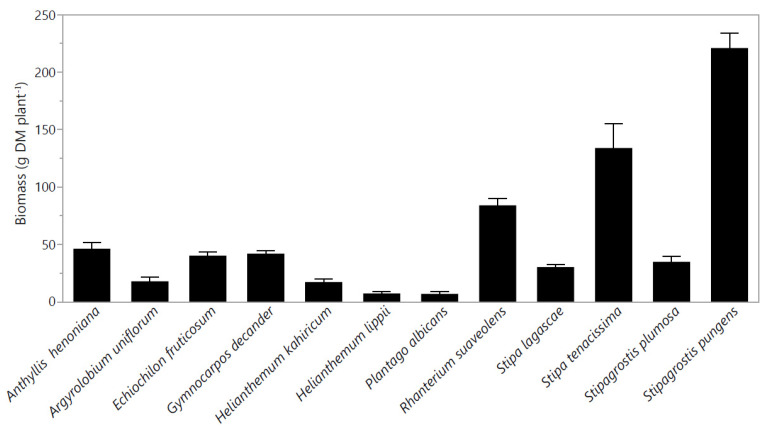
Biomass (g DM plant^−1^) of twelve native perennial species from arid rangelands of southern Tunisia. Values are the means ± standard error (SE) from 3 replicates (means ± SE).

Biomass yield varied (*p* < 0.05) among species (Figure 2). Fodder biomass ranged from 221 g DM plant^−1^ in *S. pungens* to 7 g DM plant^−1^ in *P. albicans* with an average of 56.9 g DM plant^−1^. The fodder biomass of *Stipa tenacissima* L. and *R. suaveolens* was relatively greater than the average (Figure 2).

**Figure 3:**
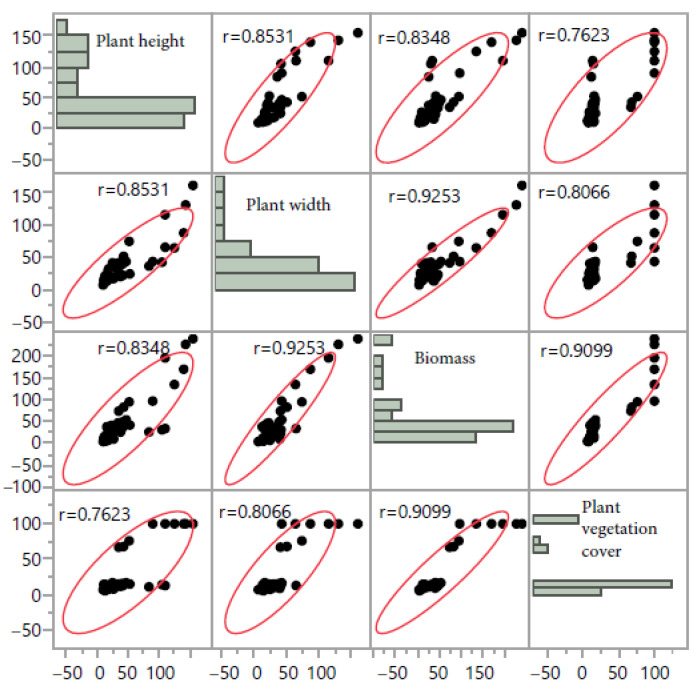
Correlation coefficients between yield attributes of twelve native perennial species from arid rangelands of southern Tunisia.

Fodder biomass was correlated with plant height (r = +83, *p* < 0.0001), plant length (r = +0.93, *p* < 0.001), and plant vegetation cover (r = +91, *p* < 0.0001; Figure 3).

### 2.2. Chemical Composition

**Figure 4:**
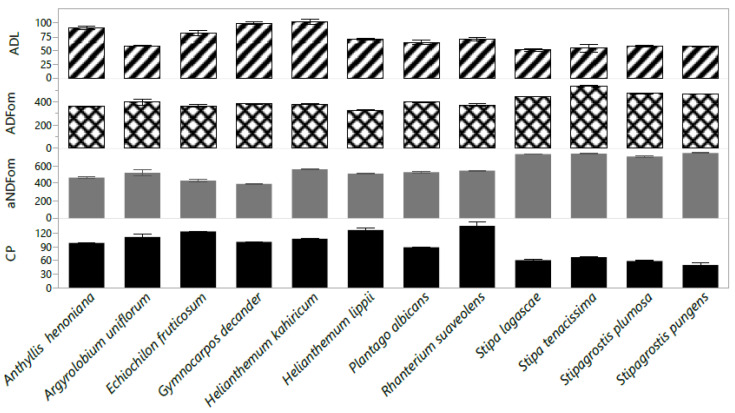
The chemical composition crude protein (CP; g/kg DM), acid detergent fiber (ADFom; g/kg DM), neutral detergent fiber (aNDFom; g/kg DM) and acid detergent lignin (ADL; g/kg DM) of twelve native perennial species from arid rangelands of southern Tunisia. Values are the means ± standard error (SE) from 3 replicates (means ± SE).

The chemical composition of the twelve species is shown in Figure 4. When compared with *Stipa* species, the foliage of *R. suaveolens*, *H. lippii*, and *E. fruticosum* species tends to be more nutritious. Considerable variation in CP concentration was observed among the species studied and ranged from 49.8 g/kg DM (*S. tenacissima*) to 13.5% (*R. suaveolens*). The greatest ADFom and aNDFom concentrations were found within species of the Poaceae family. *S. tenacissima* and *S. pungens* recorded the greatest ADFom and aNDFom (538.4; 744.5 g/kg DM), respectively. *G. decander* had the least aNDFom concentration (396.5 g/kg DM), and *H. lippii* had the least ADFom concentration (329.9 g/kg DM). Acid detergent lignin (ADL) concentration ranged between 102.4 and 50.9 g/kg DM. *H. kahiricum* had the greatest ADL concentration (102.4 g/kg DM), while low ADL concentration was recorded in species of the Poaceae family; “*S. lagascae*” (Figure 4).

**Figure 5:**
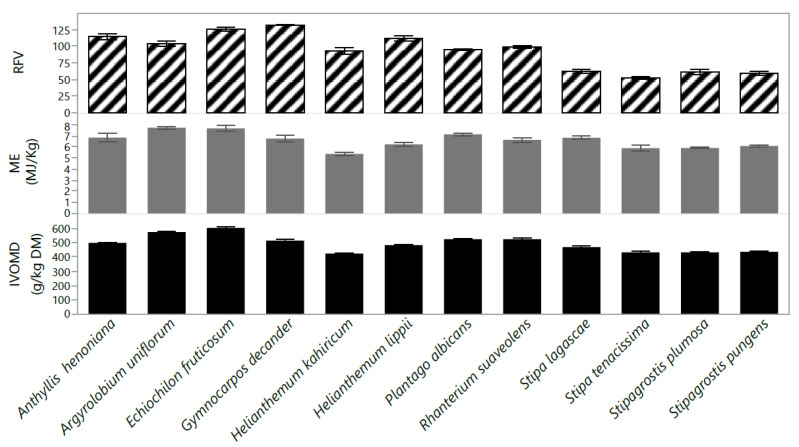
In vitro organic matter digestibility (IVOMD; g/kg DM), metabolizable energy (ME; MJ/kg) and relative feed value (RFV) of twelve native perennial species from arid rangelands of southern Tunisia. Values are the means ± standard error (SE) from 3 replicates (means ± SE).

The metabolizable energy concentration varied between 5.5 and 7.7 MJ kg DM^−1^ with the highest value for *A. uniflorum* and *E. fruticosum* and the lowest for *H. kahiricum* (Figure 5). The IVOMD of *E. fruticosum* and *A. uniflorum* was the highest (579 and 573 g kg^−1^, respectively) and lowest in *H. kahiricum* (425 g kg DM) with significant differences between species. The means of IVOMD and metabolizable energy (ME) followed a similar trend. Relative feed values (RFV) varied (*p < 0.05*) among species was lowest in *S. tenacissima* (53) and highest in *G. decander* (132). Among all species studied, *Stipa* recorded the lowest values for RFV, ranging from 53–62 (Figure 5).

**Figure 6:**
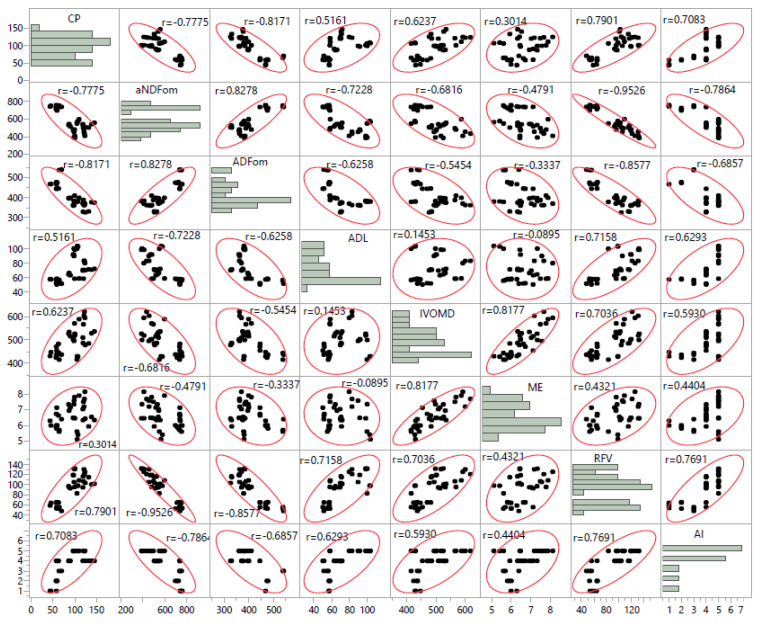
Correlation coefficients between quality attributes (CP; g/kg DM), acid detergent fiber (ADFom; g/kg DM), neutral detergent fiber (aNDFom; g/kg DM), acid detergent lignin (ADL; g/kg DM), in vitro organic matter digestibility (IVOMD; g/kg DM), metabolizable energy (ME; MJ/kg) and relative feed value (RFV), acceptability index (AI) of twelve native perennial species from arid rangelands of southern Tunisia.

There were positive and significant correlations between the IVOMD and ME (r = +0.82), and REF (r = +0.70) (Figure 6). A high negative and significant correlation was recorded between aNDFom concentration and RFV (r = −0.95). Crude protein concentrations showed a negative correlation with ADF (r = −0.82) and aNDFom (r = −0.78). Crude protein had a strong correlation with RFV (r = +0.79) and acceptability index (r = +0.71) (Figure 6).

### 2.3. Mineral Composition

Mineral concentration also varied (*p* < 0.05) among the sampled species (Table 1). *G. decander* was exceptionally high in Ca and Mg concentration (47.95 and 7.75 g/kg, respectively) and *H. lippii* contained the greatest concentration of Mn (716.2 g/kg DM). *Stipa* species showed lower levels of Ca, Mg and Mn than the other species. The Fe concentrating ranged from (mg/kg), the greatest value was in *P. albicans* and the least was in *S. lagascae*. The highest mean Cu concentrate was observed in *P. albicans* species with 107.8 g/kg DM, with a statistically significant difference (*p* < 0.05) from the other plant species except for *E. fruticosum*. The Zn ranged from 11.15 to 30.05 mg/kg DM and was lowest in *G. decander* and greatest in *S. pungens*. Na concentration varied greatly from 2.38 in *A. uniflorum* to more than 2.5 mg/kg in *R. suaveolens*.

### 2.4. Classification of Species

**Figure 7:**
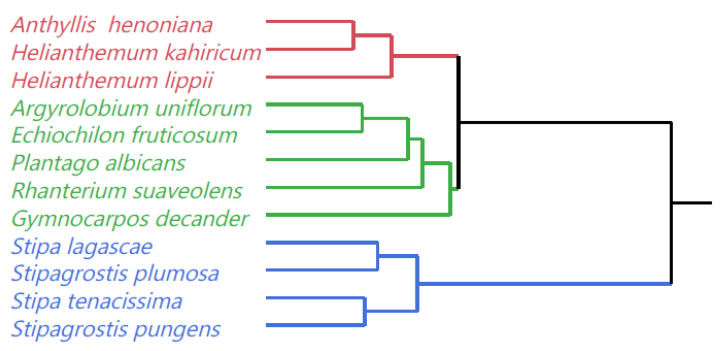
Dendrogram for twelve native perennial species from arid rangelands of southern Tunisia by average linkage method based on forage biomass yield, plant measurements and determinants of forage quality. Different colors represent different clusters (red: Cluster 1 color, green: Cluster 2: color, blue: Cluster 3).

Clusters were formed using Ward’s hierarchical clustering method and combined at each step by the method of average linkage. The data were divided into three clusters using the 21 variables measured in each species. The average CP, IVOMD, RFV, and palatability index for Cluster 1 were 42%, 8%, 4%, 8% and 50%, respectively, and greater than Cluster 3. The average species fodder biomass, height and length of Cluster 3 were more than 2.5 times that of Cluster 2. The amounts of Zn, Cu, Mn, and Na in Cluster 2 were significantly greater than in Cluster 3. In contrast, the average species fodder biomass, vegetation cover, and cell wall concentrations (ADFom and aNDFom) for Cluster 3 were significantly greater than that of Clusters 1 and 2 (Figure 7).

## 3. Discussion

The arid rangelands of southern Tunisia are dominated by perennial shrubs and grasses that serve as the major source of feed for livestock. A sound knowledge of the nutritional value of these species can be used to determine forage requirements, rangeland carrying capacity and suitable grazing time to optimize animal production while ensuring long-term vegetative cover [28,29,30,31]. The chemical analysis of range forage plants is used to measure nutritional value and mineral concentration [32]. Shrubs contained significantly greater CP than grasses, consistent with Hussain and Durrani [33], Mahmoud et al. [34], and Julian et al. [35]. *Rhanterium suaveolens*, *A. uniflorum*, and *E. fruticosum* shrubs showed high CP concentrations, while CP was lower in grasses (*Stipa* species). Compared to the other species studied and despite its low preference by grazing animals, *R. suaveolens* is a keystone species critical to rangeland structure and functioning and has important forage value [36]. The differences in CP concentration between forages may be attributed to the inherent characteristics of each species’ ability to withdraw nutrients from the soil and store them in their tissues [31,37,38]. Another reason the CP concentrations differ between species may result from differences in the accumulation of nitrogen in these forage plants during different growth periods [39]. Apart from grasses, the concentrations of CP in all other species were above 88 g/kg DM and ranged between 88–135 g/kg DM which can be classified as medium in terms of meeting sheep nutrition requirements [40].

The neutral detergent fiber (aNDFom) and the acid detergent fiber (ADFom) of grass species were higher than in other forages, which agrees with findings reported by several authors [33,41,42,43,44,45]. This is due to the fact that grasses have more stems and higher stem to leaf ratios, which results in greater concentrations of fibrous tissues compared to other forage types [31]. Generally, higher fiber concentrations result in low nutritional feed value for animals [26]. Therefore, these grass species may offer poor-quality forage compared to other species.

The higher aNDFom values within grasses were relatively close to the results reported by Megersa et al. [46], Muhakka et al. [47], and da Silva Pause et al. [48] who assessed different species of grasses in various regions. Results revealed that the mean ADFom concentration present in grasses was 460 g/kg DM, which was greater than the reported values of 326 g/kg DM and 384 g/kg DM by Katongole et al. [49] and Mosisa et al. [50]. This might be attributed to the different stages of maturity of plants at sampling [51]. Among grass species, both *S. lagascae* and *S. plumosa* appeared to have a higher palatability index and a higher nutritive value compared to other species. Both species are shorter and show lower ADFom concentrations than the other *Stipa* species. Holechek et al. [52] reported that tall grasses in general contain lower levels of nutrients than do short grasses. Unlike the aNDFom and ADFom concentrations, the ADL values tended to be greater in shrubs than grasses. These findings are in line with those of Hussain and Durrani [33] who observed higher lignin concentrations in shrubs than grasses.

Forage species with a high in vitro organic matter digestibility (IVOMD) are likely to have high nutritional value [53]. Both IVOMD and metabolizable energy (ME) are important to enhance animal performance and should be considered when preparing any feed ration. The IVOMD and ME showed a higher trend for the shrubs group compared to grasses. All studied forage species except *Stipa* and Helianthemum showed values of IVOMD above 500 g/kg DM, which is considered a good indicator that forage species have an adequate energy supply for animals [54,55]. The higher IVOMD values obtained in shrubs are within the range of 500–620 g/kg DM reported [56,57].

The ME results showed the same trend as the IVOMD. The highest ME concentration estimated for shrubs is comparable to other types of shrubs [58,59,60]. The ME depends mainly on IVOMD, which is indicated by the high positive correlation (r = +0.82) in line with Evitayani et al., [54]. The dry matter intake (DMI) is a primary factor contributing to feed efficiency and animal performance and it can be affected by the forage quality [61]. Grasses showed lower amounts of DMI and RFV compared to shrubs. This might be related to a higher aNDFom concentration that affects how much feed an animal can take in [62]. Our results confirm this finding as there was a high negative correlation between RFV and aNDFom (r = −0.95).

Minerals are essential for livestock reproductive physiology and performance due to their role in maintenance, metabolism, and growth [63,64]. In this study, mineral concentrations varied among the sampled species and the results agree with Dambe et al. [65]. The plant mineral content varies depending on species, stage of growth and environmental factors [66]. Grasses (*Stipa* spp.) had the lowest mineral concentrations. However, all other species showed Ca, Mg, Fe, Na, Mn and Cu concentration values higher than the maintenance requirements for sheep as found by Zervas [67]. Plant functional groups (grasses and shrubs) show differential mechanisms of nutrient uptake due to their contrasting root distributions, which may contribute to species coexistence [68]. On the other hand, the Zn concentration of all species was lower than the amount needed for small ruminant maintenance [69].

Our results identified three clusters. These clusters suggest that yield (shrub fodder biomass, height, and length) and fodder quality parameters could be used to identify promising forage species for rangeland rehabilitation programs. The higher fodder yield, CP, IVOMD, RFV coupled with lower values of fiber in Cluster 1 suggest that the species in this cluster could be a good option for livestock feed, especially since all the three species have a high preference index.

## 4. Materials and Methods

### 4.1. Study Area

**Figure 8:**
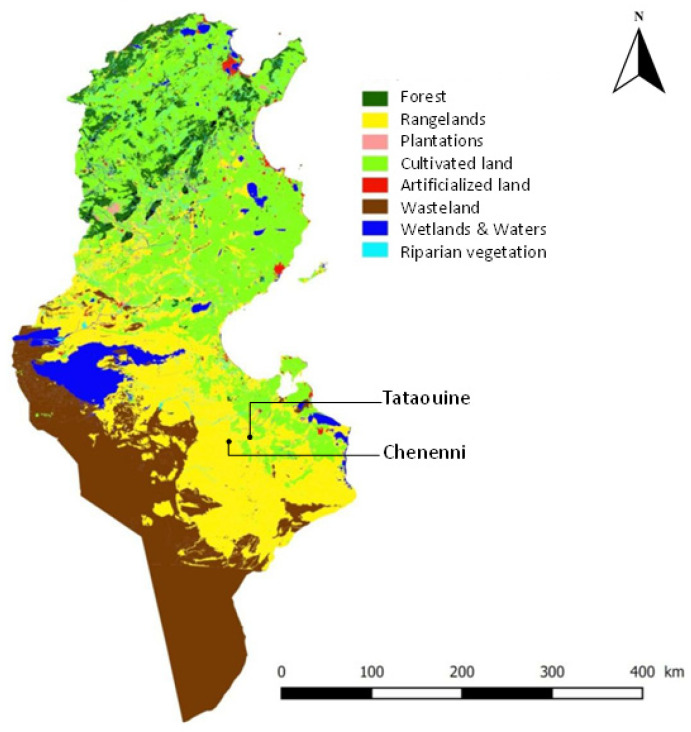
Map of Tunisia (based on land cover type [70]) showing the study site in Chenenni in the Governorate of Tataouine, Southern Tunisia.

The study area is located in the arid rangelands of Chenenni in the Governorate of Tataouine, Southern Tunisia (32°54′38.0″ N 10°15′40.2″ E, Figure 8). The climate is arid Mediterranean with a mild rainy season concentrated during autumn and spring (the growth season is from September to April) and a dry, rain-free summer lasting about four months from May to August. The landscape is dominated by villafranchian limestone crust forming undulating hills. The soil is regosol, with friable caliches at depths of 10–25 cm and gypsum outcrops.

### 4.2. Species Collection

Four grasses and eight shrub species of the most common plant species for grazing animals in the Chenenni rangelands were evaluated. Sampling collection was carried out at the flowering stage of growth during the spring of 2018. Of the 12 perennials species collected, seven were Chamaephyte and five were Hemicryptophyte. Only one of the grass species was classified as a low-acceptability species and nine as very- to high-acceptability species (Table 2).

#### 4.2.1. *Anthyllis henoniana* Coss. ex Batt.

Perennial, silky, hairy shrub species 30 to 60 cm tall belongs to the Fabaceae family. This species comes into vegetative activity after the first autumn rains. It blooms from late winter and fruiting begins in April. *A. henoniana* is a deep-rooted legume with the top fine roots found at a depth of 8 cm. *Anthyllis* takes up newly available water more rapidly than other species despite having fewer surficial roots. In areas of very low or irregular rainfall, the morphology and anatomy of *A. henoniana* have evolved to favor the interception and absorption of dew or rain directly by the shoots rather than via soil. The plant is abundant in the desert steppes mainly on calcareous and gypsum soils. It has a low presence in the arid zones but is especially common in the Saharan zones. From an edaphic point of view, steppes *A. henoniana* occupy stony and gravelly plains (Regs) that are shallow and overlain by a sandy loam, skeletal soil or by sand (sailing wind or barkanes). The plant is very palatable and is an appropriate species for the rehabilitation of degraded areas [71] and shows potential for rangeland protection against wind erosion and improvement of rangeland value [72].

#### 4.2.2. *Argyrolobium uniflorum* (Deene.) Jaub. and Spach

Perennial dwarf shrub with arcuate-ascending and densely appressed hairy stems. The plant has spreading leaves and a subterete petiole. The flowers are single, small, and have opposite leaves on short peduncles with two small, herbaceous and linear bracteoles near the middle. The legume is shortly pedunculated, densely villous-silky, torulose, dehiscent, with a persistent calyx toward the base. It produces five to seven olive-green seeds. *A. uniflorum* lives on various substrates such as limestone and sandy soils. The species lives on the upper horizon of the thermo Mediterranean belt with a semi-arid rain climate on greatly degraded soils. *A. uniflorum* is a pastoral and forage legume widely distributed in arid and semi-arid regions of Tunisia and is highly palatable and preferentially consumed by grazing animals. This plant plays an important role in the maintenance of soil fertility, soil coverage and dune stability.

#### 4.2.3. *Echiochilon fruticosum* Desf.

Small perennial shrub (10–50 cm) belongs to the Boraginaceae family. Many branches sprout from a hairy base, with the grey bark splitting to give way to reddish-brown bark. The shrub is recognizable by its sessile, pointed, thick, elongated and narrow leaves. The flowers are zygomorphic with five blue petals. *E. fruticosum* is the only species in the genus *Echiochilon* found in Tunisia. The shrub is endemic to Saharan Africa and is particularly abundant in deserts and dry rangelands on sandy ground and riverbeds of the northeast coastal areas, the center and the south and extreme south of the country. It is highly resistant to both grazing and pedo-climatic conditions. This Chamaephytic species is also among the native and keystone species historically predominant on Tunisia’s arid rangelands but currently threatened by extinction. *E. fruticosum* is known to have good nutritive value, high palatability, and is frequently foraged by small herds of sheep and goats.

#### 4.2.4. *Gymnocarpos decander* Forssk.

It is a member of the Caryophyllaceae family. A perennial undershrub 30–50 cm tall, *G. decander* is erect, suffrutescent and highly branched. The stems and branches are rough, ash grey, entangled and knotted at the nodes. The leaves are 8 to 16 mm long, 2 mm wide, obtuse, entire, mucronate and glabrous. Its flowers are sessile, pentamerous and yellowish-green and produce a one-seeded, membranous, indehiscent utricle fruit that is enclosed by persistent sepals. The seed is somewhat oblong, compressed, and dark brown with a radicle superior. *Gymnocarpos decander*, a desert plant, ideally grows among rocks and stony ridges without sand. Locally, the species is used as fuel wood and feed for grazing and therefore has economic value [73]. Because the young branches are eaten by camels and goats, plants rarely attain full growth. *Gymnocarpos decander* are palatable shrubs and are usually heavily grazed [74].

#### 4.2.5. Helianthemum kahiricum Delile

Perennial herb 15 cm tall that belongs to the Cistaceae family. It is widely distributed in the Mediterranean basin [75]. Covered by glandular hairs, the leaves are whorled, and the sessile flowers have five sepals, five yellow petals [76], numerous stamens and three to five carpels defining a unilocular ovary. The fruit is a hairy capsule. *Helianthemum kahiricum* has important pastoral, ecological, economic, and medicinal uses [77,78]. The soil habitat of this species is characterized by a moderately coarse texture, sandy loam, good water retention capacity, low organic matter concentration, basic pH and low calcium carbonate concentration. *Helianthemum kahiricum* has great potential as a forage species and is palatable to sheep, goats, and camels.

#### 4.2.6. *Helianthemum lippii* (L.) Dum. Cours.

An endemic perennial dwarf shrub belongs to the Cistaceae family. It is found in sandy regions of arid and semi-arid areas in the Mediterranean. A much-branched shrub that grows up to 60 cm tall, the branches are rigid, usually sharply tipped (in dry conditions) and whitish in appearance. Leaf length and width vary according to season (5 to 15 mm × 1 to 5 mm). The flowers are small and sessile with yellowish petals that are equal to or slightly exceed the sepals. *Helianthemum lippii* is related to desert truffles and establishes mycorrhizal symbiosis with them [79,80] and is therefore of important ecological value. *Helianthemum lippii* is one species of the genus *Helianthemum* that, according to several studies, exhibits anti-inflammatory and analgesic properties [81,82,83].

#### 4.2.7. *Plantago albicans* L.

Perennial plant belongs to the Plantaginaceae family, with a rosette hemicryptophyte form. It is recognizable by its silky, hairy aspect and lanceolate leaves with wavy margins. Its rhizomatous basis bears suckers that ensure plant survival in dry years and enables active vegetative multiplication during the growing season. The plant grows in wastelands, slopes, and stony rangelands on dry and sun-exposed soils. It colonizes open, arid parts of the Mediterranean region and runs southward in North Africa as far as sub-desertic environments. It may also occur on deep, sandy soil on surfaces of leveled silt with sharply dipping strata. *Plantago albicans* has high feed value and is collected for its various medicinal and economic uses. For instance, Plantago species have been found to possess antioxidant, antiviral, hepatoprotective, immunomodulatory, anti-inflammatory, antidiabetic, and anticancer properties [84,85,86].

#### 4.2.8. *Rhanterium suaveolens* Desf.

Perennial shrub 40 to 60 cm tall belongs to the Asteraceae family. Highly branched, the plant is also recognizable by the presence of its whitish hairs and leaves that are sessile, alternate, small, toothed, and inflorescence in capitules with ligulated, tubular yellow flowers. Endemic to the Sahara, this species is frequent in the northern region of the desert where it colonizes stabilized accumulations of sand. *Rhanterium suaveolens* dominates the shrub-steppe on sandy plains that are characterized by deep sierozem. In the period between 1975 and 2000 and despite its low palatability, the presence of *R. suaveolens* pastures on sandy soils decreased either as a result of cultivation, in particular through soil truncation, or because of overgrazing. *Rhanterium suaveolens* shrub-steppes play an important ecological role in the areas they inhabit. Its low palatability allows the plant to grow better, thus improving vegetation cover and fixing soil, which protects rangelands against desertification.

#### 4.2.9. *Stipa lagascae* R. and Sch

Perennial bunchgrass, hemicryptophyte and psammophile grass culm of 30–60 cm long. It belongs to the Poaceae family and is widely distributed throughout the Mediterranean region [87]. The flowering plant grows from the beginning of April until June [88]. It is a grazing-tolerant species but is also threatened with extinction through overgrazing [89]. It is well adapted to dry environmental conditions and can maintain growth activity during severe water deficits [90]. *Stipa lagascae* grows in sandy soils and is highly palatable for livestock. It is the most promising native grass species for land rehabilitation in arid regions [72,89,91].

#### 4.2.10. *Stipa tenacissima* L.

Perennial and rhizomatous tussock grass belongs to the Poaceae family. It has a shallow root system reaching a maximum depth of 0.5 m. Its fiber-rich leaves can reach 1 m in length. The tufts of *S. tenacissima* are circular and homogeneous when young but become empty at the center as they age and begin to die. The leaves are thin, ribbon-like, smooth, shiny and solid and are covered at the base with a hairy sheath. Esparto leaves mature in the fourth to eleventh month after budding, depending on location and climate conditions. The species is native to North Africa and is widely distributed in arid and semi-arid ecosystems of the south and western Mediterranean basin. It is a suitable species for the reclamation and rehabilitation of degraded soils as it can grow in nutrient-deficient soils, and forms a dense clump that can trap sediments and seeds and provides shelter for other species to grow. *Stipa tenacissima* is highly palatable. It is also the only raw material for making paper in Algeria, Morocco, and Tunisia and produces a fiber called esparto which is used to make cords, baskets, and espadrilles.

#### 4.2.11. *Stipagrostis plumosa* (L.) Munro ex T. Anderson

Densely caespitose, perennial desert grass belongs to the Poaceae family. It grows in dense tufts and the culms, which are erect or geniculately ascending, grow to 15 cm to 30 cm long. The lowest sheaths and internodes are covered with a thick, flocculent, and fugacious wool. The leaf blades, which are filiform, rolled, smooth, pungent, and curved, are 4 cm to 12 cm long and sometimes form a semi-circle or complete circle. The grass grows in sandy, stony, and slightly saline soils and is found in areas where the sand is stable, and also in deflation sites where it survives even when the roots and rhizosheaths are exposed. The relatively dense horizontal structure of *S. plumosa* prevents sand accumulation within the tuft. A vertical cross section through the plant has the shape of an inverted pyramid, which may act as a windbreak [92]. *Stipagrostis plumosa* is an important rangeland grass in the steppes of deserts and semi-desert regions and has high nutritive value and palatability to livestock.

#### 4.2.12. *Stipagrostis pungens* (Desf.) de Winter

Perennial grass belongs to the Poaceae family. It grows on dunes and sandy wadis. Although considered psammophyte, the species tolerates the presence of gypsum in quicksand-covered substrates. This species is much appreciated by dromedaries and to a lesser degree by small ruminants.

### 4.3. Sampling Procedure

Four For the twelve species, three plants per species with the same approximate size during the full bloom stage of the plant’s growth cycle in the spring of 2018 were selected and measured for length, width, and height. Plant cover was estimated using the supervised method in VegMeasure software (Corvallis, OR, USA). Straight-down images were taken using a Nikon Coolpix 130 digital camera (Nikon, Tokyo, Japan) with 28–140 mm zoom lens mounted to a Bogen Manfrotto 676B Monopod (Manfrotto, Cassola, Italy). Photos were taken from 1.35 m above the ground for individual plants. The dimensions of each JPG image were 4608 × 3456 pixels. A supervised classification of plant vegetative cover and soil surface was set up for images and then processed using VegMeasure software to generate processed images and summary Excel files that expressed the values (%) of the vegetative cover and soil surface in each picture [93].

Afterward, three plants of each species were sampled and pruned to one-third of the plant height above ground and separated into grazable materials (leaves plus stems less than 10 mm in diameter) and woody parts. The grazable materials were oven-dried at 60 °C for 48 h to determine dry matter concentrations for each plant.

### 4.4. Proximate Analysis

Plant samples were ground in a Thomas Model 4 Wiley mill and passed through a 1 mm sieve prior to analyses. Ash concentration was determined by burning the samples at 550 °C, and CP was determined using the Kjeldahl method. The aNDFom, ADFom, and acid detergent lignin (ADL) concentrations were determined by the sodium sulphite and alpha amylase procedure [94] and expressed exclusive of residual ash.

In vitro organic matter digestibility (IVOMD) was determined in the laboratory using the two-step procedure described by Tilley and Terry [95].

Metabolizable energy (ME) was estimated using the Menke et al. [96] equation:

ME (MJ/kg DM) = 2.2 + 0.136 G_24_ + 0.057 CP (1)

where: CP = crude Protein, G_24_ = gas production value (mL/200 mg) at 24 h.

Relative feed value (RFV) is an index used to estimate the quality of forages compared with reference feeds, i.e., alfalfa at full bloom, which is equal to 100 [97,98]. In addition to forage crops, this index has been used to estimate the quality of various and rangelands species [99,100,101]. The RFV was calculated according to Stalling [102] using the following equation:

RFV = (DMD × DMI)/1.29(2)

where: DMD = dry matter digestibility, DMI = dry matter intake, 1.29 = the expected digestible dry matter intake as % of body weight; DMI = 120/(% aNDFom)

DMD was estimated using the formula developed by Oddy et al. [103]:

DMD% = 83.58 − 0.824 ADFom% + 2.626 N%(3)

Iron (Fe), zinc (Zn), copper (Cu), manganese (Mn), sodium (Na), calcium (Ca), and magnesium (Mg) concentrations were determined by PinAAcle 900TAtomic Absorption Spectrometer (AAS).

### 4.5. Statistical Analysis

An analysis of variance was performed using the general linear model procedure of SAS (1990, SAS Institute Inc., Cary, NC, USA) to determine the differences between the nutritive values and the productive parameters. The following equation was used:

Yit = μ + τi + εit(4)

where: Yit = biomass yield or nutritional variable; μ = biomass yield or nutritional variable; τi = the effect of the rangeland’s species on the response, εit = residual error.

A univariate correlation was used to establish relationships between variables. Average values for plant height, vegetation cover, and species mineral content for each species were subjected to cluster analysis, using the Ward method to group the species into clusters. Cluster analysis and correlation were performed using SAS JMP Statistical Discovery Pro 2020 (SAS Institute Inc., Cary, NC, USA). Duncan’s Multiple Range Test was used to separate means when *p* ≤ 0.05.

## 5. Conclusions

In the arid rangelands of southern Tunisia, the production of Chamaephytes and hemicryptophytes is mainly represented by the Poaceae family, which are the dominant contributors to fodder production for livestock. The nutritive values vary widely among species but appear quite promising in energy and mineral elements for animal feeding. The moderate to high CP and low fiber concentrations along with high IVOMD found in *E. fruticosum*, *A. uniflorum*, *P. albicans*, *G. decander*, *R. suaveolens*, and *A. henoniana* suggest that these shrub species have a higher nutritive value than the highly fibrous, low IVOMD grass species *S. lagascae*, *S. tenacissima*, *S. plumosa*, and *S. pungens*. The concentration of all minerals except zinc among these shrub species was higher, suggesting that these plants are best for rangeland forages and for maintaining and enhancing livestock productivity. Therefore, it is recommended to reduce human disturbances through overgrazing or cultivation encroachment to preserve these plant communities in their natural habitats. To ensure that plant biodiversity is maintained, it will be necessary to enforce policies that ban cultivation in these fragile ecosystems and promote sustainable grazing management.

## Figures and Tables

**Table 1:** Mineral concentration (Values are the means ± standard error (SE)). of native species on arid rangelands of southern Tunisia.

Species	Fe (mg/kg DM)	Zn (mg/kg DM)	Cu (mg/kg DM)	Mn (mg/kg DM)	Na (g/kg DM)	Ca (g/kg DM)	Mg (g/kg DM)
*A. henoniana*	1690.3 ± 2.76 ^b^	15 ± 0.03 ^h^	5 ± 0.05 ^j^	31 ± 0.06 ^h^	0.6 ± 0.01 ^g^	25.8 ± 0.13 ^d^	3.1 ± 0.06 ^cd^
*A. uniflorum*	1580.7 ± 2.12 ^c^	17.6 ± 0.03 ^e^	9.6 ± 0.03 ^c^	47.7 ± 0.12 ^d^	0.2 ± 0.01 ^k^	21.3 ± 0.25 ^e^	2.7 ± 0.35 ^de^
*E. fruticosum*	543.7 ± 2.1 ^j^	15.7 ± 0.02 ^f^	10.2 ± 0.01 ^b^	70.9 ± 0.02 ^b^	1.2 ± 0.02 ^c^	26.4 ± 0.16 ^c^	1.9 ± 0.06 ^f^
*G. decander*	1129.1 ± 2.08 ^f^	11.2 ± 0.02 ^k^	5.7 ± 0.02 ^h^	33.6 ± 0.03 ^e^	1.6 ± 0.01 ^b^	48 ± 0.06 ^a^	7.8 ± 0.18 ^a^
*H. kahiricum*	1500.9 ± 2.6 ^d^	15.3 ± 0.02 ^g^	7.2 ± 0.03 ^f^	33 ± 0.05 ^g^	0.6 ± 0.02 ^f^	19.8 ± 0.16 ^f^	2.7 ± 0.12 ^cde^
*H. lippii*	1277.1 ± 2.54 ^e^	27.3 ± 0.03 ^b^	7.7 ± 0.02 ^e^	71.6 ± 0.02 ^a^	0.5 ± 0.01 ^h^	20 ± 0.09 ^f^	3.1 ± 0.06 ^c^
*P. albicans*	2106.4 ± 0.03 ^a^	21.5 ± 0.03 ^c^	10.8 ± 0.03 ^a^	54.8 ± 0.02 ^c^	0.8 ± 0.02 ^d^	28.6 ± 0.14 ^b^	4.3 ± 0.15 ^b^
*R. suaveolens*	819.9 ± 1.06 ^g^	21.3 ± 0.02 ^d^	8.9 ± 0.12 ^d^	33.3 ± 0.14 ^f^	2.5 ± 0.01 ^a^	13.6 ± 0.2 ^g^	2.5 ± 0.03 ^e^
*S. lagascae*	387 ± 1.15 ^l^	12.6 ± 0.02 ^j^	3 ± 0.02 ^k^	23 ± 0.03 ^i^	0.5 ± 0.01 ^h^	4.4 ± 0.25 ^j^	0.8 ± 0.03 ^g^
*S. tenacissima*	619.9 ± 2.06 ^h^	21.5 ± 0.02 ^c^	2.8 ± 0.01 ^l^	20.6 ± 0.01 ^j^	0.7 ± 0.01 ^e^	5.5 ± 0.15 ^i^	1 ± 0.08 ^g^
*S. plumosa*	604.1 ± 2.1 ^i,^**	14.3 ± 0.03 ^i^	5.8 ± 0.01 ^g^	18.7 ± 0.02 ^k^	0.3 ± 0.01 ^i^	6.9 ± 0.03 ^h^	1.1 ± 0.06 ^g^
*S. pungens*	537.9 ± 2.1 ^k^	30.1 ± 0.05 ^a^	5.4 ± 0.02 ^i^	17.8 ± 0.12 ^l^	0.3 ± 0.01 ^j^	6.9 ± 0.06 ^h^	1.1 ± 0.09 ^g^
Mean	1066.41	18.6	6.8	38.0	0.81	18.9	2.7
df	11	11	11	11	11	11	11
F value	143831	46084	5188	83858	4142	6755	182

** Means in a column with different letter(s) differ (*p* < 0.0001).

**Table 2:** Family, life form, and livestock acceptability index of studied species of southern Tunisia. 0: refusal or toxic; 1: occasionally palatable; 2: few palatable; 3: palatable; 4: very palatable; 5: extremely palatable.

Species	Family	Life Form	Acceptability Index
*Anthyllis henoniana* Coss. ex Batt.	Fabaceae	Chamaephyte	4
*Argyrolobium uniflorum* (Deene.) Jaub. and Spach.	Fabaceae	Chamaephyte	5
*Echiochilon fruticosum* Desf.	Boraginaceae	Chamaephyte	5
*Gymnocarpos decander* Forssk.	Caryophyllaceae	Chamaephyte	5
*Helianthemum kahiricum* Delile.	Cistaceae	Chamaephyte	4
*Helianthemum lippii* (L.) Dum. Cours.	Cistaceae	Chamaephyte	5
*Plantago albicans* L.	Plantaginaceae	Hemicryptophyte	5
*Rhanterium suaveolens* Desf.	Asteraceae	Chamaephyte	2
*Stipa lagascae* Roem. and Schult.	Poaceae	Hemicryptophyte	4
*Stipa tenacissima* L.	Poaceae	Hemicryptophyte	1
*Stipagrostis plumosa* (L.) Munro ex T. Anderson	Poaceae	Hemicryptophyte	4
*Stipagrostis pungens* (Desf.) de Winter.	Poaceae	Hemicryptophyte	3

## Data Availability

All data are available upon request.
